# Quick and Cost-Effective Estimation of Vitamin C in Multifruit Juices Using Voltammetric Methods

**DOI:** 10.3390/s20030676

**Published:** 2020-01-26

**Authors:** Jose-Antonio López-Pastor, Ascensión Martínez-Sánchez, Juan Aznar-Poveda, Antonio-Javier García-Sánchez, Joan García-Haro, Encarnación Aguayo

**Affiliations:** 1 Department of Information and Communication Technologies (TIC), Technical University of Cartagena (UPCT), ETSIT, Campus Muralla del Mar, s/n, 30202 Cartagena, Spain; 2 Postharvest and Refrigeration Group (GPR), ETSIA, UPCT, Paseo Alfonso XIII, 48, 30203 Cartagena, Spain; 3 Food Quality and Health Group. Institute of Plant Biotechnology, UPCT. Campus Muralla del Mar, s/n, 30202 Cartagena, Spain

**Keywords:** ascorbic acid, multifruit juices, electrochemical sensing, unmodified screen-printed electrodes (SPE), oxidation potential, portable low-cost potentiostat

## Abstract

Ascorbic Acid (AA) is a natural and powerful water-soluble antioxidant associated with long-lasting food products. As time passes, the AA content in products sharply decreases, and they become increasingly degraded. There are several techniques to precisely quantify AA concentrations. However, most of them employ costly laboratory instruments, such as High-Performance Liquid Chromatography (HPLC) or complex electrochemical methods, which make unfeasible recurrent AA measurements along the entire supply chain. To address this issue, we contribute with an in-field and real-time voltammetric method, carried out with a low-cost, easy-to-use, and portable device. An unmodified Screen-Printed Electrode (SPE) is used together with the device to achieve short reading times. Our method has been extensively tested in two multifruit juices using three different SPEs. Calibration curves and Limit of Detection were derived for each SPE. Furthermore, periodic experiments were conducted to study the shelf life of juices under consideration. During the analysis, a set of assays for each SPE were implemented to determine the remaining AA amount per juice and compare it with that obtained using HPLC under the same conditions. Results revealed that our cost-effective device is fully comparable to the HPLC equipment, as long as the juice does not include certain interferents; a scenario also contemplated in this article.

## 1. Introduction

Vitamin C is an essential nutrient involved in many fundamental reactions of the human body, such as the hydroxylation of proline and lysine in collagen formation or the antioxidant defence system enhancing immune functions [1,2]. In addition, high vitamin C intake has been associated with the prevention of cardiovascular diseases, as well as with low blood pressure levels [3]. Given the importance of this vital, hydrosoluble vitamin, a normal diet should contain an average amount of 91 mg per day in adults; around 110 mg for men and 78 mg for women [4]. Fruits and vegetables are our main natural source of vitamin C. Other processed products, like 100% fruit juice, could contribute to supplementing these beneficial nutrients [5]. Juices have become an important source of vitamin C in our diet, and our current style of life has promoted the ingesting of products that can be consumed “on-the-go”. However, the content of vitamin C in juices might be affected by manufacturing processes and product storage conditions [6,7].

On the other hand, the reduced form of vitamin C, ascorbic acid (AA), is widely used as a stabilizer and antioxidant in food products, increasing the shelf life of processed foods and beverages [8]. Therefore, controlling ascorbic acid content during the processing of juices and other beverages for the food industry is essential. There are several methods to determine the ascorbic acid concentration in food products, such as spectrometry [9], titrimetry [10], or high-performance liquid chromatography (HPLC) [11,12]. The HPLC method is a procedure used extensively in research and in the food industry due to its high accuracy and ability to measure both AA and the oxidized form of vitamin C, dehydroascorbic acid (DHA). All these methods share several shortcomings: high-cost; the need for specialized laboratory equipment and experienced and qualified laboratory staff; complex protocols, including tedious preparation for the extraction of vitamin C from samples; and extended analysis time for a large number of chemical reagents. From a market perspective, these concerns prevent (i) measuring the concentration of AA frequently or routinely, and (ii) accomplishing measurement campaigns outside a controlled environment, such as that provided by a laboratory. However, the food industry demands daily controls and, whenever possible, in every critical point of the production line manufacturing process. These specified requirements are related to the new regulations for quality and safety controls in the food industry by the European Commission Priorities [13].

A technique to determine ascorbic acid using novel electrochemical procedures could satisfy the demands of the food industry concerning periodicity and recurrence of the measurements. Electrochemical methods take advantage of the reductive power of bioactive elements such as ascorbic acid. The reduced form of vitamin C is an electron donor and, therefore, an electroactive substance with clear reductive properties. This fact enables us to take up the challenge of quantifying AA concentrations. Recent works have addressed this, encouraging the use of electrochemical techniques and highlighting their advantages. This interest has already been discussed by Pisoschi et al. [14], where several methods to determine AA concentration are reviewed. In particular, in the field of electrochemistry, the Screen-Printed Electrode (SPE, hereinafter) is one of the technologies that is increasingly being employed in the AA measurement of different juices. González-Sánchez et al. [15] studied SPE-based biosensors doped with ascorbate oxidase enzymes to determine AA concentrations in Pisum sativum (pea) leaves. Previously, O’Connell et al. [16] developed a sensor which altered an SPE with a conductive polyaniline (PANI) layer grown on a carbon substrate to assess the amount of AA contained in natural juices and juice concentrates. Additional intricate SPE modifications with Cu(OH)_2_ nanorods aimed at detecting AA can be found in the electrochemistry literature [17,18]. Despite the fact that SPE is a disposable, low-cost technology, modifications in its base structure, such as the ones mentioned above, improve the selectivity and sensitivity of the method, but blur the useful advantages related to their simple use, maintenance, and cost. 

Nevertheless, none of the reviewed works provide a comprehensive system together with a method able to satisfy the requirements of the food industry. Authors in [19] developed a prototype using voltammetric techniques to determine AA in standard solutions. In our study, we contribute with an innovative perspective addressed at overcoming all the abovementioned barriers. A complete method has been designed and implemented to determine AA in juices which allows (i) in-field portability, (ii) short analysis time (both aspects support the idea of determining the content of AA in juice in real time), (iii) low cost per determination, (iv) an admissible detection range for the substance under study, (v) no sample preprocessing, and (vi) no SPE modifications. In this paper, a low-cost, easy-to-use, portable electronic device is proposed and developed. 

In the present research work, in order to validate the voltammetric method to determine AA in juices, the AA content of two multifruit juices with a mixture of fruit and vegetables (orange multifruit I and red multifruit II) has been determined using three different commercial sensors made with (1) carbon electrodes (SPCE, DRP-110), (2) platinum electrodes (SPPE, DRP-550), and (3) carbon electrodes with carboxyl functionalized Multi-Walled Carbon Nanotubes (SPCE, DRP-110 CNT). The results achieved have been compared to HPLC determinations under the same conditions. Furthermore, an interference study for both multifruit juices has also been carried out to ascertain the influence of anthocyanins (one of the main sources of interference for this type of juices) in the voltammetric tests. Due to the considerable advantages of our proposed instrument, the food industry could even perform AA measurements integrated into the manufacturing process of juices, guaranteeing the quality control of their products in different phases of processing, without the need to use costly laboratory equipment or sample preprocessing. In order to carry out an analysis, the only action required is to put a controlled volume of the juice sample into the unmodified (readily available on the market) cost-effective sensor.

## 2. Materials and Methods

### 2.1. Juice Samples

In order to carry out this research, two different multifruit juices were selected. Both were manufactured and pasteurized at 88 °C for 30 s under industrial processing conditions by an international juice company. Multifruit juice I was made from orange juice (55%), white grape juice (30%), mango puree (15%); and carrot and apple juice were made from concentrate (<0.1%). Moreover, 28 mg of magnesium citrate per 100 mL of juice were added. Multifruit juice II was composed of apple juice, red grape juice, pomegranate juice, guavas, blueberries, beetroot juice, blackberry juice, and in this case, 12 mg of AA per 100 mL of juice were also supplemented. These juices were chosen for their different multiple compositions. Both juices were transported under specific refrigerated conditions from the production factory (AMC Juice and Drinks Company, Murcia, Spain) to the Postharvest and Refrigeration Group laboratory at the Technical University of Cartagena (UPCT-Cartagena, Spain). Juice I was stored for 74 days at 5 °C, and juice II was stored for 61 days at 5 °C. The quantity of vitamin C in these juices was evaluated periodically on days 7, 14, 21, 28, 45, 61, and 74 after manufacturing. Measurements in production for days 0–6 were unworkable for logistical reasons. On each sampling day, three different bottles of each juice were opened and analyzed to determine the amount of AA, using both HPLC and the proposed voltammetric method for the three different aforementioned sensors.

### 2.2. Vitamin C Analysis Using a Voltammetric Method

Three different types of screen-printed electrodes (SPEs, 4 mm ∅) were acquired from DropSens-Metrohm (Oviedo, Spain): screen-printed carbon electrodes (SPCE, DRP-110), whose counter and working electrodes are made of carbon; screen-printed platinum electrodes with high-temperature curing inks (SPPE, DRP-550) and working and counter electrodes composed of platinum; and screen-printed carbon electrodes modified with carboxyl functionalized Multi-Walled Carbon Nanotubes (SPCE, DRP-110CNT). More information regarding the morphology at microscopic level and other technical details can be found in the datasheet for each SPE [20,21,22]. All of them include a pseudo-reference electrode made of silver, and are specifically designed to use drops of quiescent samples instead of stirred solutions. In our study, these three cost-effective sensors were selected to compare their performance, since they have different working and counter electrode materials. Ascorbic acid solutions were used to calculate the calibration curves of the proposed device. They were prepared with L(+)-ascorbic acid in a solid and 99% pure form from PanReac AppliChem (ITW Reagents). 95–98% liquid sulfuric acid from Sigma-Aldrich (Merck) was also employed to reduce the instability of the ascorbic acid. All the solutions were made with deionized water obtained from a Millipore Milli-Q purification system (18.2 MΩ/cm at 25 °C).

#### 2.2.1. Field-Portable, Low-Cost Potentiostat

**Figure 1:**
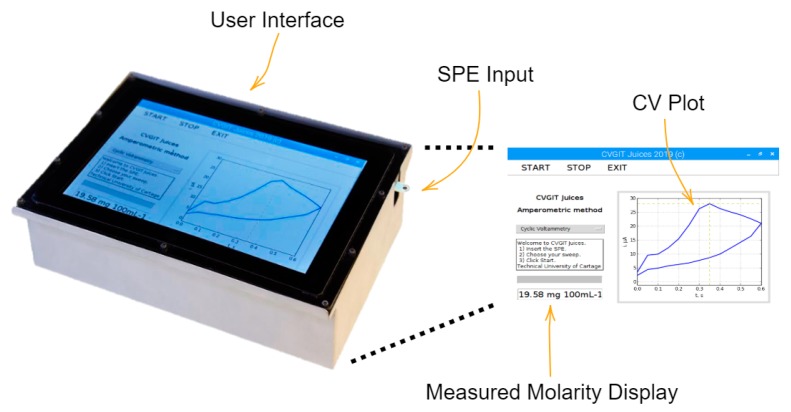
General overview of the device, and screen capture of the graphical user interface, including the plotting area of the cyclic voltammetry and the reading of ascorbic acid (AA) grams per 100 mL as a result of the analysis.

Voltammetric measurements were carried out using a portable, low-cost potentiostat device designed and implemented by integrating commercial, off-the-shelf (COTS) components. Upon obtaining and loading the calibration curves, this device later determines AA concentrations whenever it measures a sample of juice. An improved version of our previously published potentiostat [19] has been designed and developed to carry out the determination of AA in multifruit juices (Figure 1). Furthermore, note that the authors have a wide experience in this type of devices, as is corroborated in the works [23,24]. The new version of the device incorporates the following advantages: (i) full portability, allowing measurements at the industrial plant where juices or other foodstuffs are being manufactured; (ii) better isolation to reduce electromagnetic noise; and (iii) easy-to-use software (including user interface), which allows the user to put a controlled volume of the substance to be analyzed into the sensor and then simply activate a button on the touchscreen to obtain the AA level of the sample.

#### 2.2.2. Calibration of the Proposed Device

Firstly, a calibration process of the proposed device was performed for each SPE sensor (DRP-110, DRP-550, and DRP-110CNT) and for different AA standard solutions. The AA level of the reference standard ranges from 5 × 10^−5^ to 2 × 10^−3^ mM/L (0.8806–35.224 mg/100mL). Dilutions were prepared using sulfuric acid 0.5 M with type I Milli-Q® water, in order to diminish AA degradation while the measurements were taken. Additional standard solutions were prepared at pH 3.5 (the same as the multifruit juices samples) adding some drops of sulfuric acid 0.05 M to the original 0.5 M solution. Note that the AA concentrations employed in the calibration process are in the range of those contained in commercial juices (around 20–30 mg/100 mL). Concerning the calibration of the device, a 50-µL drop of standard dilution was placed on the three electrodes of the SPEs (WE, CE, and RE). Once the sample drop was poured onto the sensor, a Cyclic Voltammetry (CV) was performed from 0 to 0.6 V, with a potential step of 0.05 V/s. Taking the oxidation peak as a reference, all the points of the calibration curve were obtained. Finally, the calibration curve was derived, computing the average value of three replicas for each calibration point.

#### 2.2.3. Determination of the Limit of Detection (LoD)

The LoD was calculated from the lowest concentration of AA which differs from zero, of ten different replicas, with their standard deviation multiplied by 3.3, and this result was then divided by the calibration slope according to the procedure presented in the work by [25].

### 2.3. Vitamin C Analysis Employing HPLC

The analysis of vitamin C, as the sum of ascorbic acid (AA) and dehydroascorbic acid (DHA) content, was determined as described by [12]. The pH of the samples of refrigerated juice (25 mL) was adjusted to around 2.2–2.4 and centrifuged at 12,000× *g* for 8 min at 4 °C. The supernatant was passed through C18 Sep-Pak cartridges (Waters, Milford, MA, USA) and filtered by 0.45 µm. Standard solutions, column conditioning, and the mobile phase, wavelengths, and derivatization procedures have been previously reported by [26]. The flow rate was 1.8 mL/min. Samples of 20 μL were analyzed using HPLC equipped with an L-4200 UV–Vis detector and L-7100 pump, an AS-200A autosampler (Merck-Hitachi, Tokyo, Japan), and a Gemini-NX C18 column (250 mm × 4.6 mm; 10 μm particle size, ©Phenomenex, Inc. (Torrance, CA, USA)). The results obtained are expressed in mg per 100 g of fresh weight as the mean of three replicas per juice and time of storage.

### 2.4. Statistical Analysis

A comparison between the AA concentration obtained with the HPLC equipment and our proposed device and voltammetric method have been conducted by using the Root-mean-square deviation metric (RMSE). Concerning the correlation between both methods, a Pearson coefficient (r) and linear correlation (R2) have been also derived. These metrics were calculated using the average value of all the measurements for each day studied. In short, a good result will be achieved with a low RMSE, a slope of around 1 for the linear correlation, and a Pearson coefficient close to −1 or +1.

## 3. Results and Discussion

### 3.1. Characterization of Voltammetric Sensors

**Figure 2:**
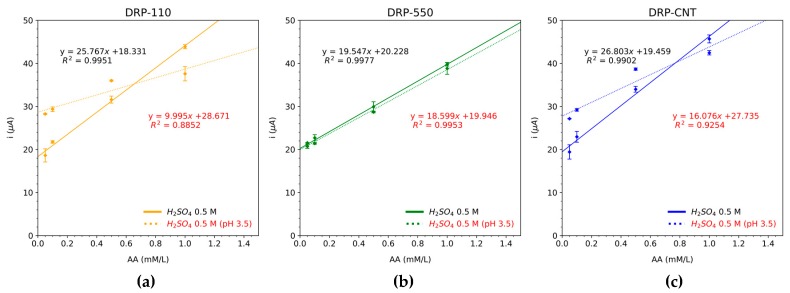
Calibration results with five concentrations in triplicate employing linear regression in a range from 0.05 to 1 mM for (i) the original standard solution of AA in H_2_SO_4_ 0.5 M and (ii) the solution adjusted to pH 3.5, for each screen-printed electrode (SPE): (**a**) DRP-110, (**b**) DRP-550, and (**c**) DRP-110CNT.

**Figure 3:**
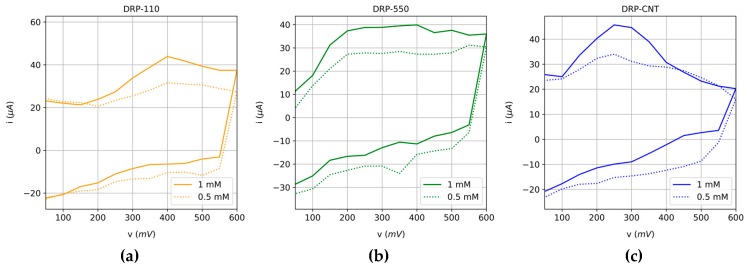
Cyclic voltammetry response of (**a**) DRP-110 sensor, (**b**) DRP-550 and (**c**) DRP-110CNT for two different AA concentrations (0.5 mM and 1 mM).

The potential at which a current peak occurs in a CV depends on different factors, such as the voltage scan rate, the chemical reactivity of the electroactive species, and the reaction rate constant (k°) [27]. All of them are intrinsically related to the ability of the electrons to flow across both the working and the counter electrodes. In particular, the larger these parameters, the greater the speed of the potential sweep and the higher the obtained current. Since AA keeps its oxidation peak at a given potential, we always sample the current at this particular potential to reduce the error of the measurements taken. This potential can be estimated from the rate constant coefficient, which, in turn, can be calculated by means of different analytical methods [28,29]. However, in the case of irreversible systems, as in the present AA case, this task becomes complex and requires advanced techniques and processing [30]. Thus, for the sake of simplicity, we have used the oxidation potential where the maximum current is achieved for the standard solution of AA. The pH is also a factor to consider because a change in its value is associated, a priori, to a change in the oxidation peak potential. Therefore, the pH was measured during all the time taken by all the tests; no significant changes were detected (pH of juices remains stable around 3.5 over time). As a result, the same potential can be used for measuring the AA degradation. These values for each type of SPE are 0.4 V for the DRP-110 and DRP-550 sensors, and 0.25 V for the DRP-110CNT. Employing the current values obtained in the potential detailed above, the calibration curve for each SPE under study is performed for two standard solutions of AA, (i) using the original solutions and (ii) adjusting the pH to the same value as the multifruit juices (3.5). Results are represented in Figure 2. Moreover, the cyclic voltammetry responses of each sensor for different molarities are illustrated in Figure 3.

According to the results shown in Figure 3, we can conclude that the three SPEs exhibit a linear response in the range of AA concentration employed in the calibration process. These results also agree with those published in previous works, where the DRP-110 is successfully used to determine the reference standard of AA [19]. The Limit of Detection (LoD) of each SPE was 0.0024 mg/100 mL, 0.0022 mg/100 mL, and 0.0210 mg/100 mL for DRP-110, DRP-550, and DRP-110 CNT, respectively.

### 3.2. Determination of Vitamin C

**Figure 4:**
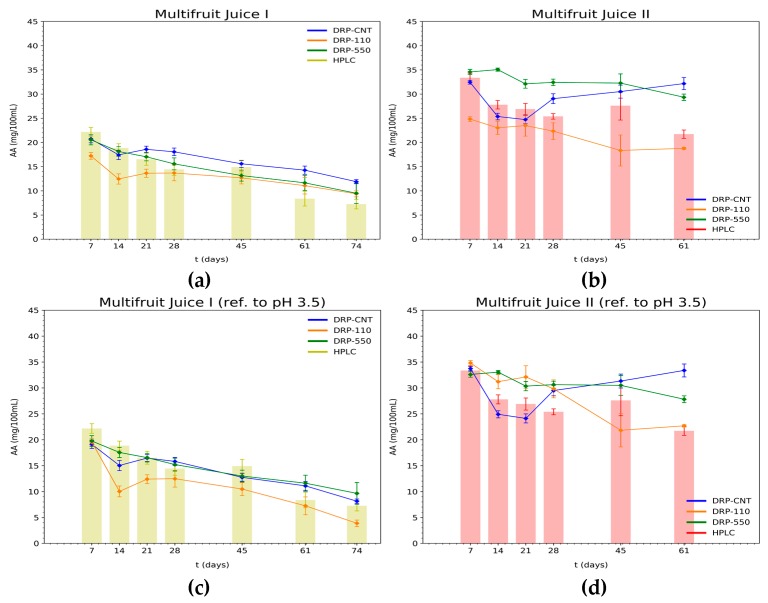
Comparison of the AA concentration achieved for each type of SPE using the proposed device and the HPLC equipment for the multifruit juices under study: orange, I (**a** and **c**); and red, II (**b** and **d**), for standard solutions at different pH. The *x*-axis represents the days of the analysis whereas the *y*-axis denotes the AA concentration.

The AA content obtained for both our proposed device and the HPLC equipment in multifruit juice I and multifruit juice II is represented in Figure 4, employing the two aforementioned standard solutions for calibration.

These results evidence the ability of our device (using unmodified electrochemical sensors) to demonstrate the decreasing tendency of AA concentration in commercial multifruit juice samples as the days of storage increase (Figure 4). Regarding the loss of AA over time, multifruit juice I suffered a significant reduction in AA content (e.g., around 62% on day 61 and 67% on day 74) compared to multifruit juice II (around 35% on day 61). These AA losses were corroborated using both the HPLC method and our system (regardless of the SPE used) for juice I; and using DRP-100 and DRP-550 sensors for juice II. Specifically, during storage, the HPLC analyses described an important reduction in AA content on days 14 and 61 in both juices. Nevertheless, different behavior was detected with our device, depending on the type of SPE and juice. In this regard, in multifruit juice I, all the sensors revealed a substantial reduction on day 14, which was more pronounced in the case of the DRP-110 sensor, and a loss of AA concentration on day 61 was also detected by the three types of sensors (Figure 4a,c). On the other hand, in multifruit juice II, the DRP-110 sensor showed two significant AA reductions on days 14 and 45, while the DRP-550 sensor also identified two relevant decreases, but later; on days 21 and 61 (Figure 4b,d). Finally, the DRP-110CNT sensor only detected a sharp decrease on day 14, exhibiting an increase in AA levels that does not correlate to the HPLC results because of the high-dispersion measurements.

### 3.3. Correlation between Voltammetric and HPLC Measuring Methods

**Figure 5:**
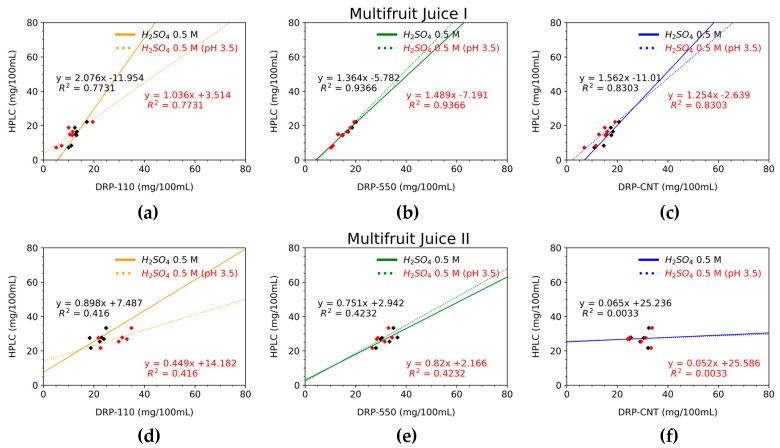
Linear correlation between the HPLC method and DRP-110 (**a** and **d**), DRP-550 (**b** and **e**), and DRP-110CNT (**c** and **f**) for juice I (**up**) and for juice II (**down**), for both standard solutions. The *x*-axis represents the AA concentration measured for each kind of SPE whereas the *y*-axis indicates the measurement employing the HPLC method.

In general, the two methods are highly correlated, and it can be said that they present a similar behavior if the slope of the correlation lines is close to 1. In Figure 5, we study this issue, correlating each screen-printed electrode response against the HPLC. Outcomes reveal that the most accurate measurements for the multifruit juice I are performed by the DRP-550 sensor, and by the DRP-110 for the multifruit juice II. As expected, the obtained slopes are not very close to 1, but bearing in mind that our proposed method is a low-cost estimation, slopes are acceptable enough. In fact, the decreasing tendency of AA in multifruit juice I was successfully detected by the DRP-550 sensor. To highlight the usefulness of our proposal in the industry, we calculate two additional metrics, the Pearson correlation and the RMSE. Results are summarized in Table 1, which compares each SPE sensor with HPLC by using the Pearson correlation and RMSE metrics. Both metrics give a clear idea of the variance and the correctness of the slope parameter. In addition, RMSE values provide the error between our method and the HPLC (note that the units are the same as the magnitude under test (mg/100 mL)). A lower value of this metric means better accuracy for certain applications and helps to decide the usefulness and applicability of the proposed method. For this scenario, the DRP-550 has the highest Pearson correlation (r = 0.9801) and the lowest RMSE (1.6442 mg/100 mL). Contrarily, multifruit juice II showed a lower correlation for all of the sensors with respect to HPLC. Despite this, the DRP-550 sensor has a higher correlation with the HPLC method than the DRP-110 or DRP-110CNT sensors (see Figure 5 and Table 1). In Table 1, we can observe that the DRP-110CNT sensor provides the lowest RMSE (5.0622 mg/100 mL); however, the Pearson coefficient indicates a low correlation (r = −0.0431). Conversely, the DRP-550 sensor has a more closely comparable RMSE than that of the DRP-110CNT (5.8858 mg/100 mL), but a better Pearson coefficient than the remaining sensors (r = 0.8457). Comparing the original calibration lines based on aqueous standard solutions in sulfuric acid 0.5M and the new ones at pH 3.5 for the multifruit I, the last one shows that the slope of the linear correlation is closer to 1 both for the DRP-110 and DRP-110CNT screen-printed electrodes, whereas the results for DRP-550 barely vary. In the case of the multifruit juice II, the new calibration results follow the trend of the first one (H_2_SO_4_ 0.5 M without pH adjustment), thus, no appreciable improvements are found due to the interferences introduced by anthocyanins.

For multifruit juice I, the correlation levels specified in Table 1 revealed the low interference of the different ingredients present in the multifruit juices. Previous research studies have shown a good correlation between HPLC and the data acquired by potentiostat of single-fruit juices. So, the AA content has been successfully quantified by the DRP-110 sensor in mono-fruit orange, grape, mango, and apple juices [31]. The AA content of carrot juices has been obtained by means of amperometric techniques in [32], resulting in a low concentration of AA. The results of these two works showed, on the one hand, that an appropriate level of AA for each component of multifruit juice I can be measured through amperometric techniques, and, on the other hand, that there are no interferences with other compounds. Outcomes comparing unmodified SPEs together with using our low-cost device against HPLC point out similarities in the decrease of AA levels over time. Additionally, Figure 5 illustrates that the DRP-550 sensor reaches the best correlation value with regard to the HPLC method (R = 0.9366). This means that AA concentrations can be appropriately determined by our potentiostat device together with this sensor, without any sample preprocessing. Regarding the DRP-110 sensor, although it is able to detect the general trend for AA loss over storage time, a specific, further adjustment is required to derive an AA concentration value as good as the one attained with the more precise and costly HPLC instrumentation.

The AA content of the different components comprising juice II has been previously quantified by amperometric methods in different works. AA in apple and guava juices was successfully determined by Jadav et al. [31]. Other fruits such as pomegranate, blueberries, and blackberries, all of them red-violet fruits, were well analyzed by Komorsky-Lovrić et al. [33] using the square-wave voltammetry technique. Blueberries were also measured by Barberis et al. [34]. All these works confirmed that the red-violet color of fruits/vegetables is provided by phenolic elements, with an important presence of anthocyanins [35]. Anthocyanins can react with dehydroascorbic acid (DHA) to reduce into AA, preventing the AA oxidation process due to their antioxidant properties, as has been previously described in the determination of L-lactate in red wine [36]. The interference of anthocyanins might account for the different reduction pattern of ascorbic acid in the multifruit juices during storage, with only an AA reduction of about 35% on day 61 of storage for juice II.

### 3.4. Interfering Compounds

As we have described above, it is worth emphasizing that there are substances that interfere with the measurements of AA content. In this section, interferences related to anthocyanins are experimentally proved. Anthocyanins, a specific kind of flavonoids (polyphenols) [37], are derived from anthocyanidins by adding sugars through the so-called glucosidic bonds. Thus, anthocyanin content is related to the content of sugar in the juice—that is, the more sugar, the greater probability of formation of anthocyanins. Based on the study of the USDA Food Data Central [38], we have pointed out on the left side of Table 2 the main compounds belonging to individual juices which compose, in turn, each multifruit juice under study (I and II). These individual juices indicated in Table 2 are sorted from left to right according to their proportion in the multifruit juices. For instance, the orange juice is in a higher proportion in multifruit juice I than the rest (grape, mango, carrot, and apple). As can be observed, sugars are one of the most abundant ingredients in the juices; this concern is key in the case of red juices since anthocyanin pigments are related to the concentration of sugar. To demonstrate that the anthocyanins are capable of interfering with the AA measurements, we performed some additional measurements employing a PGSTAT100 potentiostat/galvanostat. In particular, the cyclic voltammetry technique was applied with a very high resolution and an extended potential range up to 1.2 V. Bearing in mind that a C-18 Sep-Pak cartridge can filter the anthocyanins of the juices, we measured (i) the raw juice without filtering, (ii) the raw juice once filtered by the C-18 Sep-Pak cartridge, and (iii) the concentrated aqueous dilution of the retained anthocyanins in the cartridge.

**Figure 6:**
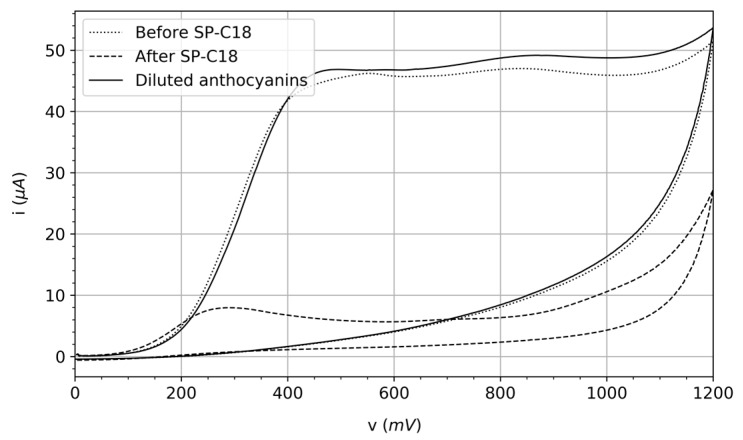
Study of discrimination of the anthocyanins extracted from the multifruit juice II.

The Figure 6 results reveal that, for the case of multifruit juice II (the red one), anthocyanins substantially interfere when intending to detect ascorbic acid. The reason is because it produces an additional current which does not correspond with the substance of interest (AA). Note that, although results for the multifruit juice (II) are clearly worse than those for the homologous multifruit juice (I); our methodology could be helpful in applications where the accuracy is not a decisive aspect, that is, applications where the instantaneous detection of abrupt drops of AA content by means of recurrent measurements is essential. In addition to these supplementary anthocyanin tests, we performed extra experiments with sugars such as glucose and fructose to discard their interference with the juices under study [39]. The response in current for both aforementioned compounds was near-zero, even for concentrations that are in accordance with those that can be found in the commercial juices.

Furthermore, we went a little further, carrying out a conscientious work to discard possible interferences in the voltammetric measurements for the different compounds of Table 2. To this end, we have collected useful information from scientific literature, in particular, the typical oxidation potential (V) for each compound, as well as the denoted Total Interference Estimation (TIE). The TIE is simply a linear estimation of the current response (µA) of the quantity of compound shown in Table 2. In this research, and based on the results represented in Figure 4, we assume that carbon, platinum, and MW-CNT electrodes provide a similar response. First, the sugar is the compound which is in higher proportion in the juices (the water is not considered in this study because it is not electroactive), followed by potassium, vitamin C, magnesium, phosphorus, calcium, sodium, and other vitamins such as B1, B2, B6, A, E, and K. According to [40], both magnesium and calcium have a negative oxidation potential, very distant from the AA one, which does not imply a significant interference. In the same way, potassium [41] and most of the vitamins, such as B2 voltammetric [42], B6 [43], A, E, and K [44], have a very different oxidation potential with respect to the AA oxidation potential. Regarding vitamin B1, it is an exception since its oxidation potential is around 0.6 V. However, the calculated TIE is very low, and the resulting interference is not remarkable either. This asseveration is justified as follows. As depicted in [44], 10·10-5 M of Vitamin B1 produces a current response around 0.8 µA. Assuming a linear behavior in all the concentration intervals, the higher amount of B1 found in juices (0.092 mg as showed in Table 2) would entail a negligible current response of around 0.31 µA. Finally, other compounds are only present in particular juices, as is the case of the β-carotene and the lutein; both are part of carrot and blackberries juices. No interferences are encountered in these latter compounds in accordance with [45].

### 3.5. Cost per Analysis

The proposed device integrates COTS components, resulting in a total cost of 150 € for the prototype version. This means that the cost of acquiring our proposed device is approximately 40 times lower than that of a professional HPLC laboratory equipment. Furthermore, by employing our system and measuring procedure, the cost per determination using DRP-550 sensors (the SPEs attaining the best performance in our work) is reduced 10 times, compared to the HPLC method. Regarding HPLC, the price stipulated here considers that the column and the reactive may be repeatedly used several times during the same day. However, note that the reactive of the column degrades in a few days, thus increasing the cost per determination. In addition to the cost per sample, portability, versatility, and the time of analysis (30 s for our solution versus 15 min in HPLC), the need of well-trained personnel to handle the HPLC equipment and equipment maintenance should be also accounted for, which is clearly higher in the chromatography instrumentation case than when employing amperometric techniques. Obviously, it should rightly be observed that HPLC is not cost-effective for measuring only one single sample; this instrument has been conceived to measure a set of continuous samples, as well as multiple substances.

## 4. Conclusions

The proposed device and associated measurement procedure enable producers to economically determine the precise AA content of multifruit juices using voltammetric methods. Our innovative solution is fully based on unmodified, disposable, commercially available SPE, coupled with a custom out-of-laboratory and cost-effective device. Its portability potentially allows for taking measurements in all the stages of the juice manufacturing process, from in-field to production lines. The tandem of SPE and device has been extensively evaluated for two different types of commercial multifruit juices, obtaining good performance in comparison with HPLC ground truth equipment. Our system is feasible and highly versatile when compared to current laboratory methods; by only putting a drop of quiescent sample in the electrodes area, AA content is determined in 30 s. However, in multifruit juices with red bioactive compounds (anthocyanins), among others, interferences in the voltammetric determination of AA could arise. Even in this case, the system is able to approximately measure AA concentration after a predefined calibration. Among the three screen-printed electrodes tested, DRP-550 produces the most accurate AA determination in multifruit juice I.

Regarding the next stages of our field-portable device, focused on achieving a nearer-to-the-market device, our future work is addressed to provide new connectivity options via Bluetooth and/or Wi-Fi interfaces, thus implementing a mobile app together with a backend and a database. In parallel, we are currently designing a custom-made electronic board to achieve a smaller, more compact, and cheaper commercial final system device.

## Figures and Tables

**Table 1:** Pearson Coefficient (r) and Root-mean-square deviation metric (RMSE) of the determination for each sensor with respect to the HPLC method employing both standard references.

Multifruit juice I					Multifruit juice II				
**Calibration**	H_2_SO_4_ 0.5 M		H_2_SO_4_ 0.5 M pH 3.5		**Calibration**	H_2_SO_4_ 0.5 M		H_2_SO_4_ 0.5 M pH 3.5	
**SPE**	Pearson Coeff. (*r*)	RMSE	Pearson Coeff. (*r*)	RMSE	**SPE**	Pearson Coeff. (*r*)	RMSE	Pearson Coeff. (*r*)	RMSE
**DRP-110**	0.8806	3.5790	0.8806	2.3323	**DRP-110**	0.6826	6.2140	0.6826	6.506
**DRP-550**	0.9801	1.6442	0.9801	2.0016	**DRP-550**	0.8457	5.8858	0.8457	4.2524
**DRP-110CNT**	0.9216	3.4315	0.9216	2.2786	**DRP-110CNT**	−0.0431	5.0622	−0.0431	5.8018

**Table 2:** AA interference study: quantity, oxidation peak, and total interference estimation (TIE) of each individual component of multifruit juices I and II.

Portion (100 mL)	Orange juice (I)	Grape juice (I)	Mango nectar (I)	Carrot juice (I)	Apple juice (I)	Pomeg-ranate juice (II)	Guavas juice (II)	Blueberries juice (II)	Root-beet juice (II)	Black-berries juice (II)	Max (I, II)	Oxidation Peak (V)	TIE (µA)	Refs.
**Sugars (g)**	9.17	14.2	12.45	3.91	10.97	1.61	13	7.92	6.64	7.7	14.2	*	*	Glucose, fructose, and linked flavonoids tested in lab. Additionally, see [39]
**Calcium (mg)**	6	11	17	24	0	11	0	8	17	14	24	*	*	[40]
**Magnesium (mg) ^1^**	10	10	3	14	0	7	0	0	0	21	14	*	*	[40]
**Potassium (mg)**	188	104	24	292	80	214	0	75	219	135	292	*	*	[41]
**Vitamin C ^2^ (mg)**	30	25	15.2	8.5	30.4	0.1	24	4.2	3.8	11.3	30.4	+0.4	-	[14,44,46]
**Thiamin (mg) B1**	0	0.017	0.003	0.092	0	0.015	0	0	0	0.012	0.092	+0.6	0.31	[44]
**Riboflavin (mg) B2**	0.028	0.015	0.003	0.055	0	0.05	0	0	0	0.018	0.055	*	*	[42,44]
**Vitamin B6 (mg)**	0.05	0.036	0.015	0.217	0	0.04	0	0	0	0.021	0.217	*	*	[43,44]
**Vitamin A (mg)**	0	0	35	956	0	0	100	0	0	6	956	*	*	[44]
**Vitamin E (mg)**	0	0	0.21	1.16	0	0.38	0	0	0	0.9	1.16	*	*	[44]
**Vitamin K (mg)**	0	0.4	0.8	15.5	0	10.4	0	0	0	15.2	15.5	*	*	[44]
**Carotene, beta (mg)**	0	5	402	9303	0	0	0	0	0	74	9303	*	*	[45]
**Lutein (mg)**	0	57	0	333	0	0	0	0	0	68	333	*	*	[45]

^1^ Magnesium concentration is higher in multifruit juice I due to its artificial enrichment. ^2^ Vitamin C (AA) concentration is higher in multifruit juice II due to its artificial addition. * Oxidation peak too far from that of AA, negligible interference.

## References

[1] Carr A., Maggini S. (2017). Vitamin C and immune function. Nutrients.

[2] Davey M.W., Van Montagu M., Inzé D., Sanmartin M., Kanellis A., Smirnoff N., Benzie I.F.F., Strain J.J., Favell D., Fletcher J. (2000). Plant L-ascorbic acid: Chemistry, function, metabolism, bioavailability and effects of processing. J. Sci. Food Agric..

[3] Padayatty S., Katz A., Wang Y., Eck P., Lee J., Chen S., Corpe C., Dutta A. (2013). Vitamin C as an antioxidant: Evaluation of its role in disease prevention vitamin C as an antioxidant: Evaluation of its role in. J. Am. Coll. Nutr..

[4] (2017). EFSA Dietary Reference Values for nutrients Summary report. EFSA Support. Publ..

[5] Crowe-White K., O’Neil C.E., Parrott J.S., Benson-Davies S., Droke E., Gutschall M., Stote K.S., Wolfram T., Ziegler P. (2016). Impact of 100% fruit juice consumption on diet and weight status of children: An evidence-based review. Crit. Rev. Food Sci. Nutr..

[6] Lee S.K., Kader A.A. (2000). Preharvest and postharvest factors influencing vitamin C content of horticultural crops. Postharvest Biol. Technol..

[7] Cilla A., Alegría A., De Ancos B., Sánchez-Moreno C., Cano M.P., Plaza L., Clemente G., Lagarda M.J., Barberá R. (2012). Bioaccessibility of tocopherols, carotenoids, and ascorbic acid from milk- and soy-based fruit beverages: Influence of food matrix and processing. J. Agric. Food Chem..

[8] Brewer M.S. (2011). Natural antioxidants: Sources, compounds, mechanisms of action, and potential applications. Compr. Rev. Food Sci. Food Saf..

[9] Wu X. (2003). Fluorimetric determination of ascorbic acid with o-phenylenediamine. Talanta.

[10] Suntornsuk L., Gritsanapun W., Nilkamhank S., Paochom A. (2002). Quantitation of vitamin C content in herbal juice using direct titration. J. Pharm. Biomed. Anal..

[11] Bassi M., Lubes G., Bianchi F., Agnolet S., Ciesa F., Brunner K., Guerra W., Robatscher P., Oberhuber M. (2017). Ascorbic acid content in apple pulp, peel, and monovarietal cloudy juices of 64 different cultivars. Int. J. Food Prop..

[12] Zapata S., Dufour J.P. (1992). Ascorbic, dehydroascorbic and isoascorbic acid simultaneous determinations by reverse phase ion interaction HPLC. J. Food Sci..

[13] European Commission Priorities: Agriculture and Food Security. https://ec.europa.eu/jrc/en/science-area/agriculture-and-food-security.

[14] Pisoschi A.M., Pop A., Serban A.I., Fafaneata C. (2014). Electrochemical methods for ascorbic acid determination. Electrochim. Acta.

[15] González-Sánchez M.I., Agrisuelas J., Valero E., Compton R.G. (2017). Measurement of total antioxidant capacity by electrogenerated iodine at disposable screen printed electrodes. Electroanalysis.

[16] O’Connell P.J., Gormally C., Pravda M., Guilbault G.G. (2001). Development of an amperometric L-ascorbic acid (Vitamin C) sensor based on electropolymerised aniline for pharmaceutical and food analysis. Anal. Chim. Acta.

[17] Reza Ganjali M. (2017). Highly sensitive voltammetric sensor for determination of ascorbic acid using graphite screen printed electrode modified with ZnO/Al_2_O_3_ nanocomposite. Int. J. Electrochem. Sci..

[18] Raveendran J., Krishnan R.G., Nair B.G., Satheesh Babu T.G. (2017). Voltammetric determination of ascorbic acid by using a disposable screen printed electrode modified with Cu(OH)_2_ nanorods. Microchim. Acta.

[19] Aznar-Poveda J., Lopez-Pastor J.A., Garcia-Sanchez A.J., Garcia-Haro J., Otero T.F. (2018). A cots-based portable system to conduct accurate substance concentration measurements. Sensors.

[20] SPCE DRP-110. http://www.dropsens.com/en/pdfs_productos/new_brochures/110-c110-c11l.pdf.

[21] SPPE DRP-550. http://www.dropsens.com/en/pdfs_productos/new_brochures/550_c550.pdf.

[22] SPCE DRP-110CNT. http://www.dropsens.com/en/pdfs_productos/new_brochures/110cnt-x1110cnt.pdf.

[23] Serafín V., Martínez-García G., Aznar-Poveda J., Lopez-Pastor J.A., Garcia-Sanchez A.J., Garcia-Haro J., Campuzano S., Yáñez-Sedeño P., Pingarrón J.M. (2019). Determination of progesterone in Saliva using an electrochemical immunosensor and a cots-based portable potentiostat. Anal. Chim. Acta.

[24] Serafín V., Arévalo B., Martínez-García G., Aznar-Poveda J., Lopez-Pastor J.A., Beltrán-Sánchez J.F., Garcia-Sanchez A.J., Garcia-Haro J., Campuzano S., Yáñez-Sedeño P. (2019). Enhanced determination of fertility hormones in saliva at disposable immunosensing platforms using a custom designed field-portable dual potentiostat. Sensors Actuators B Chem..

[25] Saadati N., Abdullah M.P., Zakaria Z., Sany S.B.T., Rezayi M., Hassonizadeh H. (2013). Limit of detection and limit of quantification development procedures for organochlorine pesticides analysis in water and sediment matrices. Chem. Cent. J..

[26] Luna M.C., Martínez-Sánchez A., Selma M.V., Tudela J.A., Baixauli C., Gil M.I. (2013). Influence of nutrient solutions in an open-field soilless system on the quality characteristics and shelf life of fresh-cut red and green lettuces (*Lactuca sativa* L.) in different seasons. J. Sci. Food Agric..

[27] Lee S.H., Fang H.Y., Chen W.C., Lin H.M., Chang C.A. (2005). Electrochemical study on screen-printed carbon electrodes with modification by iron nanoparticles in Fe(CN)6 4−/3− redox system. Anal. Bioanal. Chem..

[28] Lavagnini I., Antiochia R., Magno F. (2004). An extended method for the practical evaluation of the standard rate constant from cyclic voltammetric data. Electroanalysis.

[29] Nicholson R.S. (1965). Theory and application of cyclic voltammetry for measurement of electrode reaction kinetics. Anal. Chem..

[30] Randviir E.P. (2018). A cross examination of electron transfer rate constants for carbon screen-printed electrodes using electrochemical impedance spectroscopy and cyclic voltammetry. Electrochim. Acta.

[31] Jadav J.K., Umrania V.V., Rathod K.J., Golakiya B.A. (2018). Development of silver/carbon screen-printed electrode for rapid determination of vitamin C from fruit juices. LWT-Food Sci. Technol..

[32] Pournaghi-Azar M.H., Dastangoo H., Fadakar R. (2010). Differentiation of detection of ascorbic acid and dehydroascorbic acid using hydrodynamic amperometry and anodic stripping voltammetry on modified aluminum electrodes. Electroanalysis.

[33] Komorsky-Lovrić Š., Novak I. (2011). Abrasive stripping square-wave voltammetry of blackberry, raspberry, strawberry, pomegranate, and sweet and blue potatoes. J. Food Sci..

[34] Barberis A., Spissu Y., Fadda A., Azara E., Bazzu G., Marceddu S., Angioni A., Sanna D., Schirra M., Serra P.A. (2014). Simultaneous amperometric detection of ascorbic acid and antioxidant capacity in orange, blueberry and kiwi juice, by a telemetric system coupled with a fullerene- or nanotubes-modified ascorbate subtractive biosensor. Biosens. Bioelectron..

[35] Skrovankova S., Sumczynski D., Mlcek J., Jurikova T., Sochor J. (2015). Bioactive compounds and antioxidant activity in different types of berries. Int. J. Mol. Sci..

[36] Zanini V.P., De Mishima B.L., Labbé P., Solís V. (2010). An L-lactate amperometric enzyme electrode based on L-lactate oxidase immobilized in a laponite gel on a glassy carbon electrode. application to dairy products and red wine. Electroanalysis.

[37] Tomás-Barberán F.A. (2007). High-value co-products from plant foods: Nutraceuticals, micronutrients and functional ingredients. Handbook of Waste Management and Co-Product Recovery in Food Processing.

[38] U.S. Department of Agriculture FoodData Central. https://fdc.nal.usda.gov/.

[39] Han J.H., Choi H.N., Park S., Chung T.D., Lee W.Y. (2010). Mesoporous platinum electrodes for amperometric determination of sugars with anion exchange chromatography. Anal. Sci..

[40] Wu X.L., Zhou H.B., Wang S.J., Ye B.X. (2010). Determination of magnesium and calcium in biological samples by potentiometric stripping analysis. J. Chinese Chem. Soc..

[41] Lima A.S., Bocchi N., Gomes H.M., Teixeira M.F.S. (2009). An electrochemical sensor based on nanostructured hollandite-type manganese oxide for detection of potassium ions. Sensors.

[42] Kadara R.O., Haggett B.G.D., Birch B.J. (2006). Disposable sensor for measurement of vitamin B2 in nutritional premix, cereal, and milk powder. J. Agric. Food Chem..

[43] Brunetti B., Desimoni E. (2014). Voltammetric determination of vitamin B6 in food samples and dietary supplements. J. Food Compos. Anal..

[44] Lovander M.D., Lyon J.D., Parr D.L., Wang J., Parke B., Leddy J. (2018). Review-electrochemical properties of 13 vitamins: A critical review and assessment. J. Electrochem. Soc..

[45] Thompson G. (2016). Electrochemical Detection of Antioxidants Senior Honors. Ph.D. Thesis.

[46] Pisoschi A.M., Pop A., Negulescu G.P., Pisoschi A. (2011). Determination of ascorbic acid content of some fruit juices and wine by voltammetry performed at Pt and carbon paste electrodes. Molecules.

